# Prolonged vs intermittent intravenous infusion of β-lactam antibiotics for patients with sepsis: a systematic review of randomized clinical trials with meta-analysis and trial sequential analysis

**DOI:** 10.1186/s13613-023-01222-w

**Published:** 2023-12-05

**Authors:** Xiaoming Li, Yi Long, Guixin Wu, Rui Li, Mingming Zhou, Aiting He, Zhengying Jiang

**Affiliations:** Department of Critical Care Medicine, Chongqing University Cancer Hospital, 181 Han-Yu Road, Chongqing, 400030 China

**Keywords:** Beta-lactams, Drug administration schedule, Sepsis, Septic shock, Meta-analysis

## Abstract

**Background:**

The prolonged β-lactam antibiotics infusion has been an attractive strategy in severe infections, because it provides a more stable free drug concentration and a longer duration of free drug concentration above the minimum inhibitory concentration (MIC). We conducted this systematic review of randomized clinical trials (RCTs) with meta-analysis and trial sequential analysis (TSA) to compare the effects of prolonged vs intermittent intravenous infusion of β-lactam antibiotics for patients with sepsis.

**Methods:**

This study was prospectively registered on PROSPERO database (CRD42023447692). We searched EMBASE, PubMed, and Cochrane Library to identify eligible studies (up to July 6, 2023). Any study meeting the inclusion and exclusion criteria would be included. The primary outcome was all-cause mortality within 30 days. Two authors independently screened studies and extracted data. When the *I*^*2*^ values < 50%, we used fixed-effect mode. Otherwise, the random effects model was used. TSA was also performed to search for the possibility of false-positive (type I error) or false-negative (type II error) results.

**Results:**

A total of 4355 studies were identified in our search, and nine studies with 1762 patients were finally included. The pooled results showed that, compared with intermittent intravenous infusion, prolonged intravenous infusion of beta-lactam antibiotics resulted in a significant reduction in all-cause mortality within 30 days in patients with sepsis (RR 0.82; 95%CI 0.70–0.96; *P* = 0.01; TSA-adjusted CI 0.62–1.07). However, the certainty of the evidence was rated as low, and the TSA results suggested that more studies were needed to further confirm our conclusion. In addition, it is associated with lower hospital mortality, ICU mortality, and higher clinical cure. No significant reduction in 90-day mortality or the emergence of resistance bacteria was detected between the two groups.

**Conclusions:**

Prolonged intravenous infusion of beta-lactam antibiotics in patients with sepsis was associated with short-term survival benefits and higher clinical cure. However, the TSA results suggested that more studies are needed to reach a definitive conclusion. In terms of long-term survival benefits, we could not show an improvement.

**Supplementary Information:**

The online version contains supplementary material available at 10.1186/s13613-023-01222-w.

## Introduction

Sepsis and septic shock remain an important global health problem and a leading cause of death in critically ill patients worldwide [1, 2]. According to the Surviving Sepsis Campaign guidelines, the empirical use of broad-spectrum antibiotics against all possible pathogens immediately is recommended [3, 4]. The β-lactam antibiotics have sufficient antibacterial activity against most Gram-positive and Gram-negative bacteria, and are widely used as an important component of antibiotic therapy for patients with sepsis [5].

To our knowledge, the bactericidal activity of β-lactam antibiotics is typically time-dependent, and their clinical effectiveness is affected by the duration of the free drug concentration remains above the minimum inhibitory concentration (MIC) of the target pathogen [6]. In clinical practice, β-lactam antibiotics are typically administered via intermittent infusion. In critically ill patients, changes in renal clearance, protein binding, fluid balance and distribution volume cause large pharmacokinetic variability, leading to high variability in free drug concentration and an increased risk of underexposure to antibiotics [7, 8]. Theoretically, prolonged infusions (extended or continues infusion) can provide a more stable free drug concentration and a longer duration of free drug concentration above the MIC. Many previous studies have shown pharmacological rationale and potential clinical advantages in favor of prolonged intravenous infusion of β-lactam antibiotics in critically ill patients [9]. Therefore, based on “moderate-quality” evidence, the latest Surviving Sepsis Campaign guidelines suggest a “weak” recommendation for prolonged infusion of β-lactam antibiotics for patients with sepsis or septic shock, rather than conventional bolus infusion [10].

Prolonged infusion strategies have many advantages. However, compared with intermittent infusion strategies, prolonged infusion strategies require special infusion pumps and infusion bags that are costly. Moreover, some β-lactam antibiotics (e.g., meropenem) are not stable under prolonged exposure at room temperature and may produce and enhance degradation products that cause hypersensitivity reactions [11]. The true clinical benefits of prolonged infusion strategies are still debated. Most of prior studies have been limited due to low study quality. Kondo and colleagues performed a systematic review and meta-analysis included 13 studies showed that prolonged intravenous infusion of β-lactam antibiotics for patients with sepsis or septic shock did not reduce in-hospital mortality compared to intermittent intravenous infusion [12]. Another systematic review and meta-analysis involving 22 studies reported prolonged intravenous infusion was associated with lower all-cause mortality [13]. Recently, Mirjalili and colleagues published a study reported prolonged intravenous infusion can significantly reduce hospital mortality, ICU mortality, and improve clinical cure [14]. However, the largest randomized clinical trial (RCT) on this issue published in JAMA recently found prolonged intravenous infusion did not improve clinically relevant outcomes [15].

Based on published RCTs, we performed this systematic review of RCTs with meta-analysis and trial sequential analysis (TSA) to compare the clinical efficacy of prolonged vs intermittent intravenous infusion of β-lactam antibiotics for patients with sepsis.

### Methods

We performed this study following the Preferred Reporting Items for Systematic Reviews and Meta-Analyses (PRISMA statement) guidelines and using Review Manager (version 5.3) [16]. In addition, this study was prospectively registered on PROSPERO database (Registration number: CRD42023447692).

### Search strategy and eligibility criteria

We searched EMBASE, PubMed, and the Cochrane Library from inception through to July 6, 2023. The search strategy was decided by our team, which the keywords and free-text words related to sepsis, beta-lactam antibiotics, and drug administration schedule were used. The search strategy details for each database are shown in Additional file 1.

The inclusion criteria were as follows: (1) population: adult patients (≥ 18 years) diagnosed with sepsis received β-lactam antibiotics for infection; (2) intervention: the study group patients received β-lactam antibiotics by prolonged infusion strategy (extended or continuous); (3) comparison: the control group received β-lactam antibiotics by intermittent infusion strategy; (4) outcome: all-cause mortality within 30 days or clinical cure were reported; (5) study design: RCT; and (6) language: published in English. The exclusion criteria were as follows: (1) study not focused on sepsis or septic shock; (2) the type of antibiotics was different between the two groups; and (3) study only reported pharmacodynamic or pharmacokinetic data, and neither all-cause mortality within 30 days nor clinical cure was available. All identified studies were evaluated by two authors (X.L and Y.L) independently. Any disagreements between two authors were resolved through discussion. In case of persistent disagreement, we consulted the third reviewer (Z.J) for arbitration.

### Data extraction and outcomes

The following data were extracted by two authors (X.L and Y.L) in prespecified forms and checked by a third author (Z.J): the first author, year of publication, study period, sample size (male/female), details of antibiotic infusion strategy (type, dose, and duration), and all clinical outcomes.

The primary outcome was all-cause mortality within 30 days. If 28-day mortality or 30-day mortality was reported, it was used for analysis. If not, hospital mortality or ICU mortality would be used. The secondary outcomes included clinical cure, duration of treatment, emergence of resistance bacteria, length of ICU stay, length of hospital stay, 90-day all-cause mortality, hospital all-cause mortality, ICU all-cause mortality.

### Statistical analysis

For binary outcomes, we reported the risk ratios (RRs) and 95% confidence intervals (CIs). Continuous outcomes were pooled by calculating the mean differences (MDs) with a 95% CI. When necessary, median and interquartile range (IQR) were converted to means and standard deviation by methods described by Wan et al. [17]. Heterogeneity among the included studies was assessed using the *I*^*2*^ statistic [18]. When the *I*^*2*^ values < 50%, we used the fixed-effect mode. Otherwise, the random-effect model was used as appropriate. If the two-sided *p* value was less than 0.05, the results were considered statistically significant. We did subgroup analyses for the primary outcome based on doses of antibiotic (equeal in two groups or not equal in two groups). We performed sensitivity analyses for the primary outcome by excluding one study without using of a loading dose in the prolonged infusion group. We did sensitivity analyses for duration of treatment, length of ICU stay and length of hospital stay by excluding studies that did not report the mean or standard deviation of these outcomes. Pubication bias was assessed by Funnel plots and Egger test.

### Trial sequential analysis

TSA was performed to test if the meta-analysis had reached the required information size and to address potential issues in meta-analyses such as insufficient statistical power to detect intervention effects, which can lead to false-positive (type I error) or false-negative (type II error) results [19]. We used a random effects model to construct the cumulative Z curve. TSA was performed to maintain an overall 5% risk of a type I error. Based on previous high-quality RCTs on this topic [15, 20], we used an anticipated relative risk reduction (RRR) of 12.5% with a power of 80% to calculate the required information size to detect or reject an intervention effect. And the control event rate was set as 30% according to previous review [1].

### Risk of bias assessment and GRADE approach

We evaluated the risk of bias for each of these studies by the Cochrane risk of bias assessment tool, which included following items: the random sequence generation, allocation concealment, blinding of participants and personnel, blinding of outcome assessment, incomplete outcome data, selective reporting, and other bias [21]. In addition, we overall graded the evidence for each finding in this study by the Grading of Recommendations Assessment, Development, and Evaluation (GRADE) tool, which classifies the body of evidence as high, moderate, low, and very low four categories [22]. The above assessment was done independently by two authors (X.L and Y.L). Any disagreements were resolved by discussion, if no agreement could be reached, it would be decided by a third author (Z.J).

## Results

### Selection of included studies

**Fig. 1:**
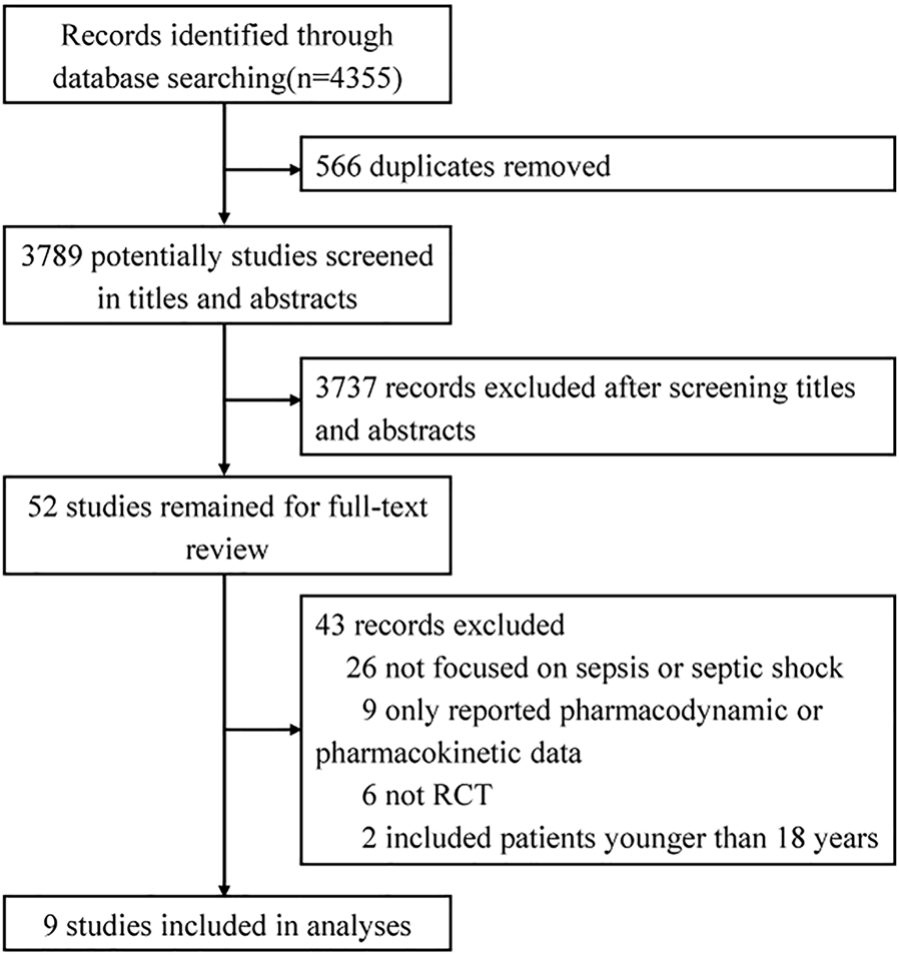
Flow diagram for the identification of eligible studies

**Fig. 2:**
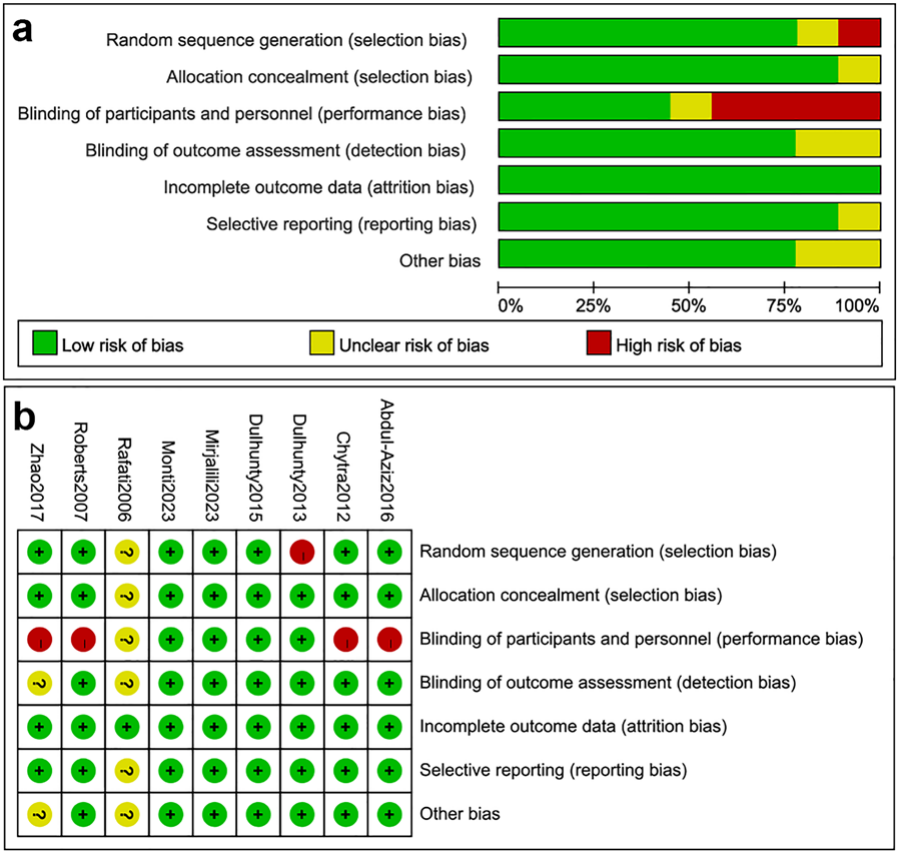
**a** Risk of bias graph; **b** Risk of bias summary graph

A total of 4355 studies were identified in our search. According to the inclusion and exclusion criteria, only nine studies with 1762 patients were finally included in this meta-analysis [14, 15, 20, 23–28]. The process of study selection is presented in Fig. 1. Table 1 summarizes the characteristics of the included individual studies. The studies were published from 2006 to 2023. The number of participants ranged from 40 to 607. Three studies were multicenter studies [15, 24, 25], two were two-center studies [14, 20], and four were single-center studies [23, 26–28]. The definition of sepsis or septic shock varied from study to study. In most of studies, antibiotics were limited to just one type of β-lactam antibiotic, but three studies provided three types of β-lactam antibiotics for doctor to choose [20, 24, 25]. The predominantly β-lactam antibiotic in the reviewed studies was meropenem, which was included in six studies [15, 20, 23–25, 28]. For the prolonged infusion group, only one study adopted extended infusing strategy [14], and all other studies adopted continues infusion strategy. Five studies had a high risk of bias, four for performance bias and one for selection bias [20, 23, 24, 27, 28] (Fig. 2).

**Table 1:** Characteristics of included studies

Study	Design and enrollment period	Participants (number/male/age)		Sepsis definition	Antibiotics	Loading dose	Prolonged infusion	Intermittent infusion
		Prolonged group	Intermittent group					
Monti^15^ (2023)	Multicenter June 2018 to August 2022	303/195 65.5 ± 14.0	304/209 63.4 ± 15.0	Sepsis-3, traditional definitions	Meropenem	Yes	3 g over 24 h, continuous	1 g every 8 h, over 30 to 60 min
Mirjalili^14^ (2023)	Two centers August 2019 to August 2021	68/37 53.82 ± 15.82	68/38 53.10 ± 16.22	NA	Ampicillin–sulbactam	Yes	9 g every 8 h, over 4 h	9 g every 8 h, over 30 min
Zhao^28^ (2017)	Single center June 2012 to December 2014	25/10 68.0 ± 15.4	25/11 67.0 ± 12.2	Sepsis-2	Meropenem	Yes	3 g over 24 h, continuous	1 g every 8 h, over 30 min
Abdul-Aziz^20^ (2016)	Two centers April 2013 to July 2014	70/46 54 (42–63)	70/50 56 (41–68)	Sepsis-2	Meropenem, piperacillin–tazobactam, cefepime	Yes	3 g over 24 h, 18 g over 24 h, 6 g over 24 h, continuous	1 g every 8 h, 4.5 g every 6 h, 2 g every 8 h, over 30 min
Dulhunty^25^ (2015)	Multicenter July 2012 to April 2014	212/130 64 (54–72)	220/135 65 (53–72)	Sepsis-2	Meropenem, piperacillin–tazobactam, ticarcillin–clavulanate	Yes	3 g over 24 h, 13.5 g over 24 h, 12.4 g over 24 h, continuous	1 g every 8 h, 4.5 g every 8 h, 3.1 g every 6 h, over 30 min
Dulhunty^24^ (2013)	Multicenter April 2010 to November 2011	30/23 54 ± 19	30/19 60 ± 19	Sepsis-2	Meropenem, piperacillin–tazobactam, ticarcillin–clavulanate	No	Continuous, clinician-chosen	Bolus, clinician-chosen
Chytra^23^ (2012)	Single center September 2007 to May 2010	120/78 44.9 ± 17.8	120/83 47.2 ± 16.3	Sepsis-2	Meropenem	Yes	4 g over 24 h, continuous	2 g every 8 h, over 30 min
Roberts^27^ (2007)	Single center NA	29/16 43 ± 19	28/17 52 ± 16	SIRS criteria	Ceftriaxone	Yes	2 g over 24 h, continuous	2 g every 24 h, bolus
Rafati^26^ (2006)	Single center October 2003 to March 2004	20/12 50.1 ± 22.2	20/15 48.0 ± 20.7	Sepsis-2	Piperacillin	Yes	8 g over 24 h, continuous	3 g every 6 h, over 30 min

*NA* not available; *SIRS* systemic inflammatory response syndrome

### Mortality

**Fig. 3:**
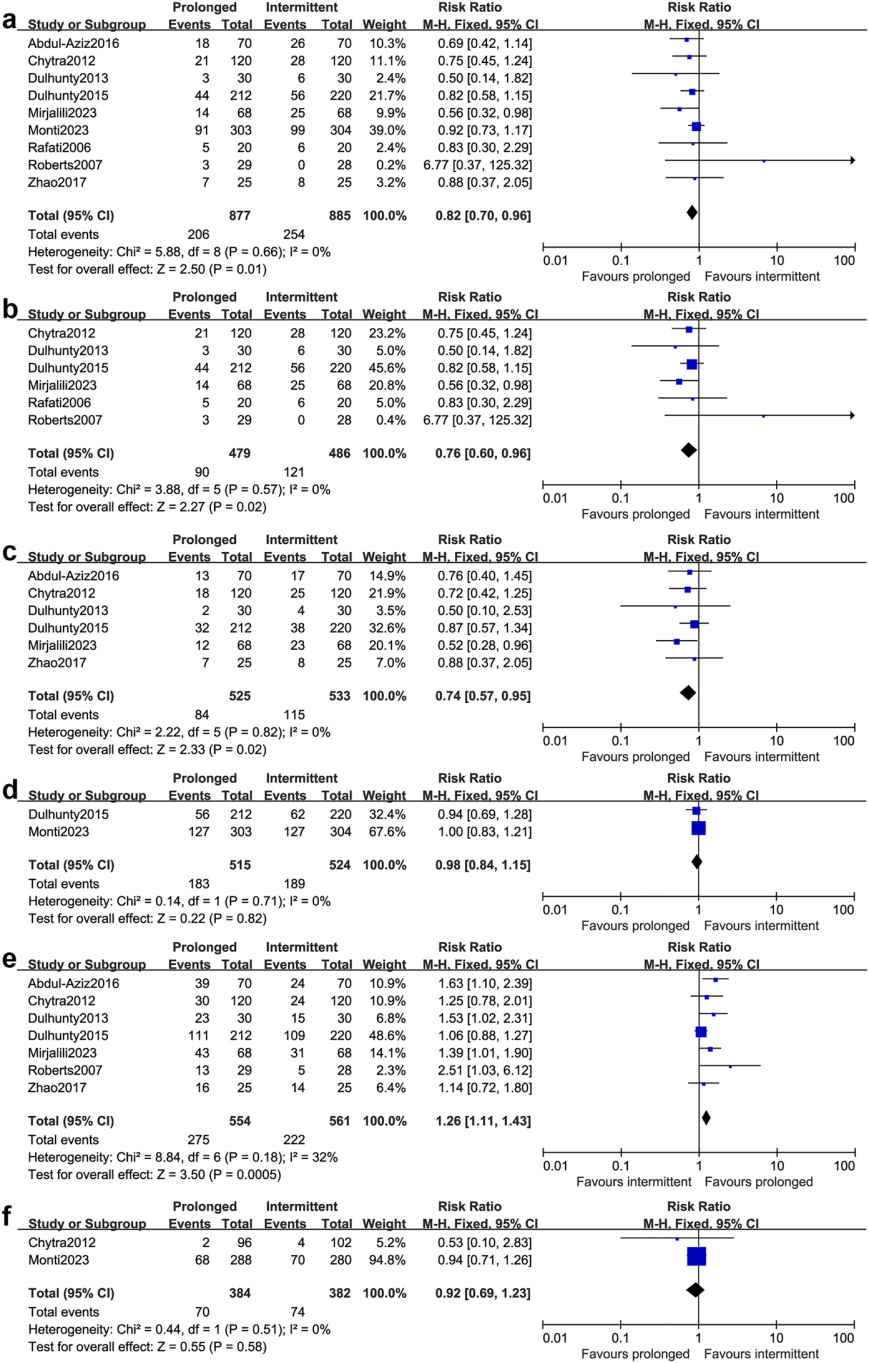
Forest plot of comparison: prolonged infusion group vs intermittent infusion group. **a** All-causes mortality within 30 days; **b** Hospital mortality; **c** ICU mortality; **d** 90-day mortality; **e** Clinical cure; **f** Emergence of resistance bacteria

**Fig. 4:**
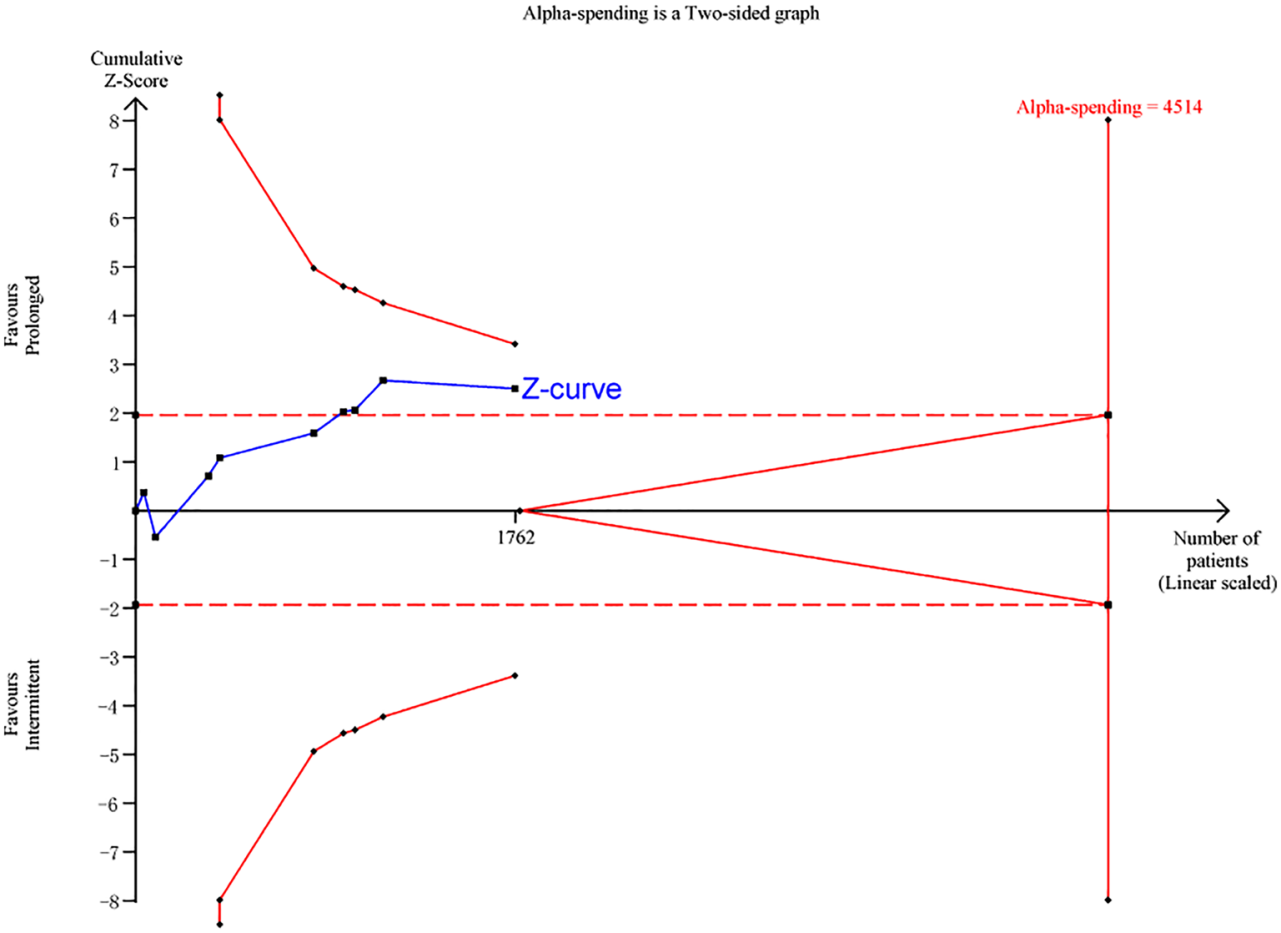
Trial sequential analysis. The cumulative Z-curve (complete blue line) was constructed using a random effect model. Etched red line shows conventional test boundary. Complete red line represents the trial sequential monitoring boundary. A diversity-adjusted information size of 4514 patients were calculated based on using alfa = 0.05 (two sided), beta = 0.20 (power 80%), an anticipated relative risk reduction (RRR) of 12.5%, and a control event rate of 30.0%. The cumulative Z-curve crossed conventional test boundary; however, it did not cross Alpha-spending boundary, nor did it reach the required information size

All studies provided data for all-cause mortality within 30 days at different endpoints (one reported 30-day mortality, one reported 28-day mortality, six reported hospital mortality, one reported ICU mortality). Overall, mortality in the prolonged group and the intermittent group were 23.5% (206 of 877 patients) and 28.7% (254 of 885 patients), respectively. The pooled results showed that the prolonged intravenous infusion of β-lactam antibiotics for patients with sepsis could significantly decrease in all-cause mortality within 30 days compared with intermittent intravenous infusion (RR 0.82; 95%CI 0.70–0.96; *P* = 0.01; Fig. 3a). No significant heterogeneity among studies was not detected (*I*^*2*^ = 0%;* P* = 0.66; Fig. 3a). No significant publication bias was detected by funnel plots and Egger test(*P* = 0.086; see Additional file 2). TSA result showed that the required information size was 4514. The cumulative Z-curve crossed conventional test boundary; however, it did not cross Alpha-spending boundary, nor did it reach the required information size (Fig. 4). Due to the accrued information size was too small compared to the required information size, the TSA-adjusted CI becomes wider than the traditional 95% CI (TSA-adjusted CI 0.62–1.07). It is suggested that the result may have the possibility of false positive and more studies are required.

The pooled results showed that the prolonged intravenous infusion of β-lactam antibiotics for patients with sepsis could also significantly decrease in hospital mortality [14, 23–27] (RR 0.76; 95% CI 0.60–0.96; *P* = 0.02; *I*^*2*^ = 0%; 965 participants, six studies, Fig. 3b) and ICU mortality [14, 20, 23–25, 28] (RR 0.74; 95% CI 0.57–0.95; *P* = 0.02; *I*^*2*^ = 0%; 1058 participants, six studies, Fig. 3c) compared with intermittent intravenous infusion. However, only two studies reported 90-day mortality and the result showed no significant difference between the two groups [15, 25] (RR 0.98; 95% CI 0.84–1.15; *P* = 0.82; *I*^*2*^ = 0%; 1039 participants, Fig. 3d).

### Clinical cure and duration of treatment

**Fig. 5:**
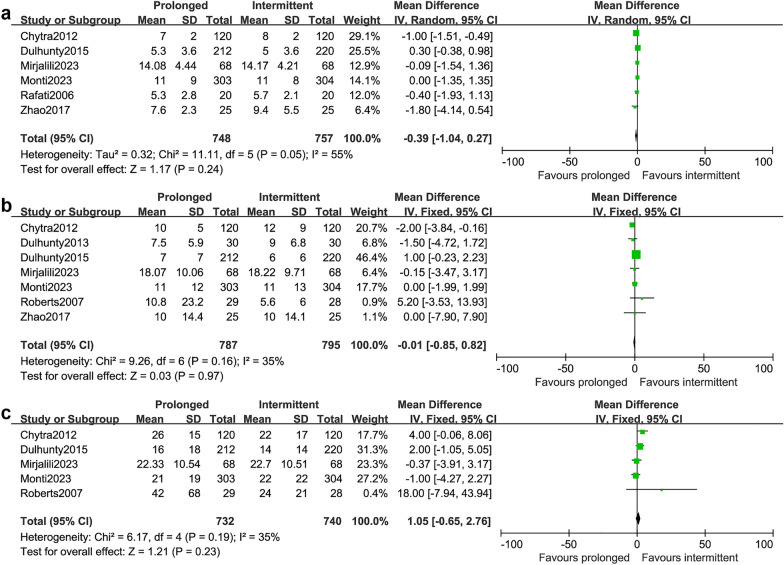
Forest plot of comparison: prolonged infusion group vs intermittent infusion group. **a** Duration of treatment; **b** The length of ICU stays, **c** The length of hospital stays

Seven studies involving 1115 patients reported the clinical cure [14, 20, 23–25, 27, 28]. The clinical cure was mainly defined as complete disappearance of all signs and symptoms related to infection. In four studies [14, 23, 27, 28], the time for evaluation was at completion of treatment. However, in the other three studies, one was at 7–14 days after study drug cessation [24], and two was at 14 days [20, 25]. The result indicated that the prolonged infusion strategy could significantly improve clinical cure (RR 1.26; 95% CI 1.11–1.43; *P* = 0.0005; *I*^*2*^ = 32%; Fig. 3e). Data of duration of treatment were available in six studies involving 1505 patients [14, 15, 23, 25, 26, 28]. The result indicated that the prolonged infusion strategy was not associated with shorter duration of treatment compared with the intermittent infusion strategy (MD -0.39; 95% CI -1.04–0.27; *P* = 0.24; *I*^*2*^ = 55%; Fig. 5a).

### Length of ICU stay and length of hospital stay

Seven studies involving 1582 patients reported the length of ICU stay [14, 15, 23–25, 27, 28] and five studies involving 1472 patients reported the length of hospital stay [14, 15, 23, 25, 27]. The pooled results showed that the prolonged infusion strategy did not shorten the length of ICU stay (MD -0.01; 95% CI -0.85–0.82; *P* = 0.97; I^2^ = 35%; Fig. 5b), nor did it shorten the length of hospital stay (MD 1.05; 95% CI -0.65–2.76; *P* = 0.23; I^2^ = 35%; Fig. 5c).

### Emergence of resistance bacteria

Only two studies had available data on emergence of resistance bacteria [15, 23]. In the study conducted by Chytra et al. [23], the time frame for assessing the emergence of resistant bacteria was from treatment initiation to the end of therapy. In the study conducted by Monti et al. [15], the emergence of resistant bacteria was assessed at 28 days after randomization. There were 70 patients (18.2%) who were detected with resistance bacteria among 384 patients in the prolonged infusion group and 74 patients (19.4%) who were detected with resistance bacteria among 382 patients in the intermittent infusion group. No significant difference was found (RR 0.92; 95% CI 0.69–1.23; *P* = 0.58; I^2^ = 0%; Fig. 3f).

### Subgroup analyses and sensitivity analyses

From the subgroup analyses of the primary outcome, we found that when the total daily antibiotic dose was consistent between the two groups, the prolonged infusion strategy was superior to the intermittent infusion strategy [14, 15, 20, 25, 27, 28] (RR 0.84; 95% CI 0.71–0.99; *P* = 0.04; *I*^*2*^ = 3%; Additional file 3a). However, when the total daily antibiotic dose was inconsistent between the two groups, there was no significant difference [23, 26] (RR 0.76; 95% CI 0.49–1.20; *P* = 0.25; *I*^*2*^ = 0%; Additional file 3b). We conducted sensitivity analyses for primary outcome by excluding one study without using of a loading dose in the prolonged infusion group [24], and the conclusion was consistent with the main analysis (RR 0.83; 95% CI 0.71–0.97; *P* = 0.02; *I*^*2*^ = 0%; Additional file 3c). The conclusions of sensitivity analyses for duration of treatment (MD − 0.29; 95% CI − 1.16–0.58; *P* = 0.51; I^2^ = 0%; Additional file 3d), length of ICU stay (MD 0.53; 95% CI − 2.58–3.63; *P* = 0.74; I^2^ = 21%; Additional file 3e) and length of hospital stay (MD − 0.03; 95% CI − 3.54–3.47; *P* = 0.98; I^2^ = 47%; Additional file 3f) were also consistent with the main analyses.

### Certainty of the evidence

Due to the definition of sepsis or septic shock existed inconsistency among studies, we downgraded the quality of evidence for all results by one level. For the primary outcome, if 28-day mortality or 30-day mortality was not reported, we used hospital mortality or ICU mortality for analysis. Therefore, we downgraded the quality of evidence by one level due to indirectness. For the length of ICU stay, length of hospital stay, and duration of treatment, data from some studies were expressed in terms of median and IQR. Therefore, we downgraded the quality of evidence for these results by one level due to imprecision. Although the risk of bias was existed in some studies, most information is from studies at low or unclear risk of bias. In addition, we thought potential limitations are unlikely to lower confidence in the estimate of effect. Therefore, the final assessment of risk of bias was not serious and we did not downgrade the certainty of the evidence in the GRADE assessment due to the risk of bias. Finally, the certainty of the evidence of the primary outcome, the length of ICU stay, the length of hospital stay, and duration of treatment was rated as low, and the certainty of the evidence of other outcomes was rated as moderate (Table 2).

**Table 2:** Summary of findings table

Outcomes	Anticipated absolute effects* (95% CI)		Relative effect (95% CI)	No. of participants (studies)	Certainty of the evidence (GRADE)
	Risk with intermittent infusion	Risk with Prolonged infusion			
All-cause mortality within 30 days	287 per 1,000	235 per 1000 (201 to 276)	RR 0.82 (0.70 to 0.96)	1762 (9 RCTs)	⨁⨁◯◯ Low ^a,b^
Hospital mortality	249 per 1,000	189 per 1000 (149 to 239)	RR 0.76 (0.60 to 0.96)	965 (6 RCTs)	⨁⨁⨁◯ Moderate ^a^
ICU mortality	216 per 1,000	160 per 1000 (123 to 205)	RR 0.74 (0.57 to 0.95)	1058 (6 RCTs)	⨁⨁⨁◯ Moderate ^a^
90-day mortality	361 per 1,000	353 per 1000 (303 to 415)	RR 0.98 (0.84 to 1.15)	1039 (2 RCTs)	⨁⨁⨁◯ Moderate ^a^
Clinical cure	396 per 1,000	499 per 1000(439 to 566)	RR 1.26 (1.11 to 1.43)	1115 (7 RCTs)	⨁⨁⨁◯ Moderate ^a^
Emergence of resistance	194 per 1,000	178 per 1000 (134 to 238)	RR 0.92 (0.69 to 1.23)	766 (2 RCTs)	⨁⨁⨁◯ Moderate ^a^
Duration of treatment		MD 0.39 lower (1.04 lower to 0.27 higher)		1505 (6 RCTs)	⨁⨁◯◯ Low ^a,c^
Length of ICU stay		MD 0.01 lower (0.85 lower to 0.82 higher)		1582 (7 RCTs)	⨁⨁◯◯ Low ^a,c^
Length of hospital stay		MD 1.05 higher (0.65 lower to 2.76 higher)		1472 (5 RCTs)	⨁⨁◯◯ Low ^a,c^

GRADE Working Group grades of evidence

High certainty: we are very confident that the true effect lies close to that of the estimate of the effect

Moderate certainty: we are moderately confident in the effect estimate: the true effect is likely to be close to the estimate of the effect, but there is a possibility that it is substantially different

Low certainty: our confidence in the effect estimate is limited: the true effect may be substantially different from the estimate of the effect

Very low certainty: we have very little confidence in the effect estimate: the true effect is likely to be substantially different from the estimate of effect

^*^The risk in the intervention group (and its 95% confidence interval) is based on the assumed risk in the comparison group and the relative effect of the intervention (and its 95% CI)

*CI* confidence interval; *RR* risk ratio; *MD* mean difference; *ICU* intensive care unit

^a^Downgraded the quality of evidence one level for inconsistency

^b^Downgraded the quality of evidence one level for indirectness

^c^Downgraded the quality of evidence one level for imprecision

## Discussion

This meta-analysis showed that, compared with intermittent intravenous infusion, prolonged intravenous infusion of beta-lactam antibiotics resulted in a significant reduction in all-cause mortality within 30 days in patients with sepsis. However, the certainty of the evidence was rated as low, and the TSA results suggested that more studies were needed to further confirm our conclusion. In addition, it is associated with lower hospital mortality, ICU mortality, and higher clinical cure. No significant reduction in 90-day mortality or the emergence of resistance bacteria was detected between the two groups, which may be due to fewer studies providing data on 90-day mortality and emergence of resistance bacteria.

We updated systematic review and meta-analysis on this issue with the addition of two recently published high-quality RCTs [14, 15]. To avoid the obvious heterogeneity caused by the large difference of the study population, we only included studies focusing on sepsis or septic shock. The results of this study are consistent with most previous similar studies showing that prolonged intravenous infusion of beta-lactam antibiotics in patients with severe infection are associated with improved clinical outcomes [12, 13, 29–31]. In a meta-analysis of individual patient data from RCTs, it seemed that patients with an Acute Physiology and Chronic Health Evaluation II (APACHE II) score of 22 or higher benefited more from prolonged infusion than patients with an APACHE II score of less than 22, including hospital mortality, ICU mortality, and clinical cure [31]. This can be explained by the fact that the more severe the disease, the more obvious changes in pharmacokinetic/pharmacodynamic parameters are due to various reasons [32, 33]. In addition, in theory, prolonged infusion is more likely to achieve pharmacokinetic/pharmacodynamic targets than intermittent infusion [8].

Unfortunately, because some related data were not available, we were not able to further validate these results with subgroup analyses based on disease severity scores, such as APACHE II scores and Sequential Organ Failure Assessment (SOFA) scores.

The conclusion of our study appears to be inconsistent with those of the Continuous Infusion vs Intermittent Administration of Meropenem in Critically Ill Patients (MERCY) trial [15], the biggest RCT on this issue to date. In the MERCY trial, 91 of 303 patients (30%) in the continuous administration group died within 28 days, compared with 99 of 304 patients (33%) in the intermittent administration group. Although no significant difference was found between two groups (RR 0.92; 95% CI 0.73–1.17; *P* = 0.50), the absolute 28-day mortality in the continuous administration group was lower than that in the intermittent administration group. Thus, adding data from the MERCY trial to our meta-analysis would support rather than refute the previously reported mortality benefits [34]. Currently, many studies have shown short-term survival benefits with prolonged infusion, but only two studies have reported 90-day mortality and found no long-term survival benefits [15, 25]. In the study conducted by Monti et al., the 90-day mortality was 42% in both groups, while in the study of Dulhunty et al., the 90-day mortality were 26% in the continuous group and 28% in the intermittent group, which was significantly lower than the results of Monti et al. This may be due to differences in the definition of sepsis and disease severity between studies. In our study, the 90-day mortality was 35.5% (183 of 515 patients) in the prolonged infusion group and 36.1% (189 of 524 patients) in the intermittent infusion group. No significant difference was found between two groups (RR 0.98; 95% CI 0.84–1.15; *P* = 0.82), which is consistent with previous RCTs. The ongoing study aimed at comparing the effects of continuous infusion of β-lactam antibiotics (piperacillin–tazobactam or meropenem) vs intermittent infusion on 90-day mortality in critically ill patients with sepsis will shed light on long-term survival benefits [35].

The β-lactam antibiotics are the most commonly used antibacterial agents, accounting for more than half of global antibiotic use. However, with the emergence of β-lactam resistant bacteria, the clinical therapeutic application of β-lactam antibiotics is threatened [5]. Prevention of resistance is also an important goal in our treatment process [36]. In critically ill patients, unpredictable pharmacokinetics are often caused by organ perfusion changes and dysfunction, which often results in inadequate antibiotic exposure, potentially reducing efficacy and promoting the emergence of resistance bacteria [7, 37]. In general, prolonged infusion of β-lactam antibiotics can provide stable serum levels, improve their efficacy, and to some extent decrease the emergence of antimicrobial resistance. Our study shows that the prolonged intravenous infusion strategy can significantly improve clinical cure, but do not with significantly reduce the emergence of resistance bacteria. Only two studies reported the emergence of resistant bacteria in this meta-analysis [15, 23]. However, the incidence of resistant bacteria was significant difference. That may be due to the time frame for assessing the emergence of resistant bacteria and the definition of emergence of resistant bacteria existed difference between studies. More studies and uniform evaluation criteria are required to test whether prolonged intravenous infusion strategy could decrease the emergence of antimicrobial resistance. According to the subgroup analysis, we found that although the total dose of antibiotics in the intermittent infusion group was higher than that in the prolonged infusion group, it did not reduce mortality (RR 0.76; 95% CI 0.49–1.20; *P* = 0.25), suggesting that prolonged infusion may avoid unnecessary excessive antibiotic exposure.

Notwithstanding the TSA results suggested that more studies were needed to further confirm our conclusion, clinical practice may favor prolonged infusion strategies given the ongoing possibility of benefit and absence of harm. For future research, we have the following considerations: first, we should combine pharmacokinetic and pharmacodynamic results with clinical outcomes, which can further elucidate the potential mechanism of the influence of different infusion strategies on clinical outcomes; second, subgroup analyses should be performed based on the disease severity, which may identify which populations are likely to benefit more from prolonged infusion strategies; and third, a cost-effectiveness analysis is necessary, which is also an important aspect we need to consider when choosing different infusion strategies.

Our meta-analysis has several limitations. The main limitation was the inconsistent of definition of sepsis in the included studies. Therefore, we downgraded the quality of evidence for all results by one level. Second, to avoid the obvious heterogeneity caused by the large difference of the study population, we only included studies focusing on sepsis or septic shock. The results applied to other situations should be cautious. Third, due to limited data, we did note perform subgroup analysis by severity of disease, types of antibiotics, etc. Finally, long-term survival benefits require additional studies to further evaluate.

## Conclusions

Prolonged intravenous infusion of beta-lactam antibiotics in patients with sepsis was associated with short-term survival benefits and higher clinical cure. However, the TSA results suggested more studies are needed to reach a definitive conclusion. In terms of long-term survival benefits, we could not show an improvement.

### Supplementary Information

**Additional file 1. **Search strategy terms and results.

**Additional file 2. ** a. Funnel plots; b. Egger test.

**Additional file 3. **Forest plot of the subgroup analyses and sensitivity analyses: a. Subgroup analyses for the primary outcome based on doses of antibiotic (equal in two groups); b. subgroup analyses for the primary outcome based on doses of antibiotic (not equal in two groups); c. sensitivity analyses for the primary outcome by excluding one study without using of a loading dose in the prolonged infusion group; d. sensitivity analyses for duration of treatment; e. Sensitivity analyses for length of ICU stay; f. Sensitivity analyses for length of hospital stay.

## Data Availability

All data generated or analyzed during this study are included in this published article [and its additional information files].

## Abbreviations

APACHE II: Acute Physiology and Chronic Health Evaluation II
CIs: Confidence intervals
GRADE: Grading of Recommendations Assessment, Development, and Evaluation
ICU: Intensive Care Unit
IQR: Interquartile range
MDs: Mean differences
MIC: Minimum inhibitory concentration
PRISMA: Preferred Reporting Items for Systematic Reviews and Meta-Analyses
RCTs: Randomized clinical trials
RRs: Risk ratios
RRR: Relative risk reduction
SOFA: Sequential Organ Failure Assessment
TSA: Trial sequential analysis

## References

[1] Bauer M, Gerlach H, Vogelmann T, Preissing F, Stiefel J, Adam D (2020). Mortality in sepsis and septic shock in Europe, North America and Australia between 2009 and 2019- results from a systematic review and meta-analysis. Crit Care.

[2] Rudd KE, Johnson SC, Agesa KM, Shackelford KA, Tsoi D, Kievlan DR, Colombara DV, Ikuta KS, Kissoon N, Finfer S (2020). Global, regional, and national sepsis incidence and mortality, 1990–2017: analysis for the global burden of disease study. Lancet.

[3] Dellinger RP, Rhodes A, Evans L, Alhazzani W, Beale R, Jaeschke R, Machado FR, Masur H, Osborn T, Parker MM (2023). Surviving sepsis campaign. Crit Care Med.

[4] Levy MM, Evans LE, Rhodes A (2018). The surviving sepsis campaign bundle: 2018 update. Crit Care Med.

[5] Mora-Ochomogo M, Lohans CT (2021). beta-Lactam antibiotic targets and resistance mechanisms: from covalent inhibitors to substrates. RSC Med Chem.

[6] Craig WA (1998). Pharmacokinetic/pharmacodynamic parameters: rationale for antibacterial dosing of mice and men. Clin Infect Dis.

[7] Roberts JA, Paul SK, Akova M, Bassetti M, De Waele JJ, Dimopoulos G, Kaukonen KM, Koulenti D, Martin C, Montravers P (2014). DALI: defining antibiotic levels in intensive care unit patients: are current beta-lactam antibiotic doses sufficient for critically ill patients?. Clin Infect Dis.

[8] Veiga RP, Paiva JA (2018). Pharmacokinetics-pharmacodynamics issues relevant for the clinical use of beta-lactam antibiotics in critically ill patients. Crit Care.

[9] Abdul-Aziz MH, Portunato F, Roberts JA (2020). Prolonged infusion of beta-lactam antibiotics for gram-negative infections: rationale and evidence base. Curr Opin Infect Dis.

[10] Evans L, Rhodes A, Alhazzani W, Antonelli M, Coopersmith CM, French C, Machado FR, McIntyre L, Ostermann M, Prescott HC (2021). Surviving sepsis campaign: international guidelines for management of sepsis and septic shock 2021. Intensive Care Med.

[11] Abdul-Aziz MH, Dulhunty JM, Bellomo R, Lipman J, Roberts JA (2012). Continuous beta-lactam infusion in critically ill patients: the clinical evidence. Ann Intensive Care.

[12] Kondo Y, Ota K, Imura H, Hara N, Shime N (2020). Prolonged versus intermittent beta-lactam antibiotics intravenous infusion strategy in sepsis or septic shock patients: a systematic review with meta-analysis and trial sequential analysis of randomized trials. J Intensive Care.

[13] Vardakas KZ, Voulgaris GL, Maliaros A, Samonis G, Falagas ME (2018). Prolonged versus short-term intravenous infusion of antipseudomonal beta-lactams for patients with sepsis: a systematic review and meta-analysis of randomised trials. Lancet Infect Dis.

[14] Mirjalili M, Zand F, Karimzadeh I, Masjedi M, Sabetian G, Mirzaei E, Vazin A (2023). The clinical and paraclinical effectiveness of four-hour infusion vs half-hour infusion of high-dose ampicillin-sulbactam in treatment of critically ill patients with sepsis or septic shock: an assessor-blinded randomized clinical trial. J Crit Care.

[15] Monti G, Bradic N, Marzaroli M, Konkayev A, Fominskiy E, Kotani Y, Likhvantsev VV, Momesso E, Nogtev P, Lobreglio R (2023). Continuous vs intermittent meropenem administration in critically ill patients with sepsis: the MERCY randomized clinical trial. JAMA.

[16] Liberati A, Altman DG, Tetzlaff J, Mulrow C, Gotzsche PC, Ioannidis JP, Clarke M, Devereaux PJ, Kleijnen J, Moher D (2009). The PRISMA statement for reporting systematic reviews and meta-analyses of studies that evaluate health care interventions: explanation and elaboration. PLoS Med.

[17] Wan X, Wang W, Liu J, Tong T (2014). Estimating the sample mean and standard deviation from the sample size, median, range and/or interquartile range. BMC Med Res Methodol.

[18] Higgins JP, Thompson SG, Deeks JJ, Altman DG (2003). Measuring inconsistency in meta-analyses. BMJ.

[19] Brok J, Thorlund K, Gluud C, Wetterslev J (2008). Trial sequential analysis reveals insufficient information size and potentially false positive results in many meta-analyses. J Clin Epidemiol.

[20] Abdul-Aziz MH, Sulaiman H, Mat-Nor MB, Rai V, Wong KK, Hasan MS, Abd Rahman AN, Jamal JA, Wallis SC, Lipman J (2016). Beta-lactam infusion in severe sepsis (BLISS): a prospective, two-centre, open-labelled randomised controlled trial of continuous versus intermittent beta-lactam infusion in critically ill patients with severe sepsis. Intensive Care Med.

[21] Higgins JP, Altman DG, Gotzsche PC, Juni P, Moher D, Oxman AD, Savovic J, Schulz KF, Weeks L, Sterne JA (2011). The Cochrane Collaboration's tool for assessing risk of bias in randomised trials. BMJ.

[22] Balshem H, Helfand M, Schunemann HJ, Oxman AD, Kunz R, Brozek J, Vist GE, Falck-Ytter Y, Meerpohl J, Norris S (2011). GRADE guidelines: 3 rating the quality of evidence. J Clin Epidemiol.

[23] Chytra I, Stepan M, Benes J, Pelnar P, Zidkova A, Bergerova T, Pradl R, Kasal E (2012). Clinical and microbiological efficacy of continuous versus intermittent application of meropenem in critically ill patients: a randomized open-label controlled trial. Crit Care.

[24] Dulhunty JM, Roberts JA, Davis JS, Webb SA, Bellomo R, Gomersall C, Shirwadkar C, Eastwood GM, Myburgh J, Paterson DL (2013). Continuous infusion of beta-lactam antibiotics in severe sepsis: a multicenter double-blind, randomized controlled trial. Clin Infect Dis.

[25] Dulhunty JM, Roberts JA, Davis JS, Webb SA, Bellomo R, Gomersall C, Shirwadkar C, Eastwood GM, Myburgh J, Paterson DL (2015). A multicenter randomized trial of continuous versus intermittent beta-lactam infusion in severe sepsis. Am J Respir Crit Care Med.

[26] Rafati MR, Rouini MR, Mojtahedzadeh M, Najafi A, Tavakoli H, Gholami K, Fazeli MR (2006). Clinical efficacy of continuous infusion of piperacillin compared with intermittent dosing in septic critically ill patients. Int J Antimicrob Agents.

[27] Roberts JA, Boots R, Rickard CM, Thomas P, Quinn J, Roberts DM, Richards B, Lipman J (2007). Is continuous infusion ceftriaxone better than once-a-day dosing in intensive care? A randomized controlled pilot study. J Antimicrob Chemother.

[28] Zhao HY, Gu J, Lyu J, Liu D, Wang YT, Liu F, Zhu FX, An YZ (2017). Pharmacokinetic and pharmacodynamic efficacies of continuous versus intermittent administration of meropenem in patients with severe sepsis and septic shock: a prospective randomized pilot study. Chin Med J.

[29] Kiran P, Nadir Y, Gencer S (2023). Clinical efficacy and safety of prolonged versus intermittent administration of antipseudomonal beta-lactam antibiotics in adults with severe acute infections: a meta-analysis of randomized controlled trials. J Infect Chemother.

[30] Rhodes NJ, Liu J, O'Donnell JN, Dulhunty JM, Abdul-Aziz MH, Berko PY, Nadler B, Lipman J, Roberts JA (2018). Prolonged infusion piperacillin-tazobactam decreases mortality and improves outcomes in severely ill patients: results of a systematic review and meta-analysis. Crit Care Med.

[31] Roberts JA, Abdul-Aziz MH, Davis JS, Dulhunty JM, Cotta MO, Myburgh J, Bellomo R, Lipman J (2016). Continuous versus intermittent beta-lactam infusion in severe sepsis. A meta-analysis of individual patient data from randomized trials. Am J Respir Crit Care Med.

[32] Roberts JA, Abdul-Aziz MH, Lipman J, Mouton JW, Vinks AA, Felton TW, Hope WW, Farkas A, Neely MN, Schentag JJ (2014). Individualised antibiotic dosing for patients who are critically ill: challenges and potential solutions. Lancet Infect Dis.

[33] Venuti F, Trunfio M, Martson AG, Lipani F, Audagnotto S, Di Perri G, Calcagno A (2023). Extended and continuous infusion of novel protected beta-lactam antibiotics: a narrative review. Drugs.

[34] Shappell CN, Klompas M, Rhee C (2023). Do prolonged infusions of beta-lactam antibiotics improve outcomes in critically Ill patients with sepsis?. JAMA.

[35] Lipman J, Brett SJ, De Waele JJ, Cotta MO, Davis JS, Finfer S, Glass P, Knowles S, McGuinness S, Myburgh J (2019). A protocol for a phase 3 multicentre randomised controlled trial of continuous versus intermittent beta-lactam antibiotic infusion in critically ill patients with sepsis: BLING III. Crit Care Resusc.

[36] Drusano GL (2003). Prevention of resistance: a goal for dose selection for antimicrobial agents. Clin Infect Dis.

[37] Pereira JG, Fernandes J, Duarte AR, Fernandes SM (2022). beta-lactam dosing in critical patients: a narrative review of optimal efficacy and the prevention of resistance and toxicity. Antibiotics.

