# Supplementary material for: Prolonged vs intermittent intravenous infusion of β-lactam antibiotics for patients with sepsis: a systematic review of randomized clinical trials with meta-analysis and trial sequential analysis
Source: Ann Intensive Care. 2023 Dec 5;13:121. doi: 10.1186/s13613-023-01222-w (PMC10697919; doi:10.1186/s13613-023-01222-w)
Supplement: Supplementary file 1 — Additional file 1. Search strategy terms and results. [file 13613_2023_1222_MOESM1_ESM.docx]

Additional file 1. Search Strategies in the systematic review

1. PubMed search strategy（July 6, 2023）

| #1 | Sepsis [MeSH Terms]) OR Shock, Septic [MeSH Terms]) OR "Systemic inflammatory response syndrome" [MeSH Terms]) OR "Bacterial Infections" [MeSH Terms]) OR Sepsis [Title/Abstract]) OR Septic* [Title/Abstract]) OR Infection [MeSH Terms])) | 3,147,827 |
| --- | --- | --- |
| #2 | (beta-Lactams [MeSH Terms] OR carbapenem* [Title/Abstract] OR penicillin* [Title/Abstract] OR piperacillin [Title/Abstract]) OR cephalosporin* [Title/Abstract]) OR meropenem [Title/Abstract]) OR imipenem [Title/Abstract]) OR doripenem [Title/Abstract]) OR ticarcillin [Title/Abstract]) OR cefepime [Title/Abstract]) OR ceftazidime [Title/Abstract]) OR cefoperazone [Title/Abstract]) OR monobactam [Title/Abstract]) OR aztreonam [Title/Abstract])) OR Ertapenem [Title/Abstract]) OR Cefazolin [Title/Abstract]) OR "Clavulanic Acid*"[Title/Abstract]) OR Sulbactam [Title/Abstract]) OR Tazobactam [Title/Abstract])) OR beta-Lactam* [Title/Abstract]) | 215,964 |
| #3 | (Drug Administration Schedule [MeSH Terms]) OR (extended [Title/Abstract]) OR prolonged [Title/Abstract]) OR continuous [Title/Abstract]) OR intermittent [Title/Abstract]) | 1,289,528 |
| #4 | Administration, Intravenous [MeSH Terms] OR infusion* [Title/Abstract]) OR administration* [Title/Abstract] | 1,208,438 |
| #5 | #3 AND #4 | 142,705 |
| #6 | #1 AND #2 AND #5 | 3,046 |
| #7 | ((“randomized controlled trial” [pt] OR “controlled clinical trial” [pt] OR randomized [tiab] OR placebo [tiab] OR “drug therapy” [sh] OR randomly [tiab] OR trial [tiab] OR groups [tiab])) | 5,776,563 |
| #8 | #6 AND #7 | 1,962 |

2. The Cochrane Central Register of Controlled Trials search strategy（July 6, 2023）

| #1 | MeSH descriptor: [Sepsis] explode all trees | 6770 |
| --- | --- | --- |
| #2 | MeSH descriptor: [Shock, Septic] explode all trees | 1249 |
| #3 | MeSH descriptor: [Systemic Inflammatory Response Syndrome] explode all trees | 7339 |
| #4 | MeSH descriptor: [Infection] explode all trees | 100180 |
| #5 | (sepsis):ti,ab,kw | 13315 |
| #6 | (septic*):ti,ab,kw | 6487 |
| #7 | (bacteremia):ti,ab,kw | 2875 |
| #8 | (infect*):ti,ab,kw | 155126 |
| #9 | #1 OR #2 OR #3 OR #4 OR #5 OR #6 OR #7 OR #8 | 199833 |
| #10 | MeSH descriptor: [beta-Lactams] explode all trees | 10523 |
| #11 | (carbapenem*):ti,ab,kw OR (penicillin*):ti,ab,kw OR (piperacillin):ti,ab,kw OR (cephalosporin*):ti,ab,kw OR (meropenem):ti,ab,kw | 7801 |
| #12 | (imipenem):ti,ab,kw OR (doripenem):ti,ab,kw OR (ticarcillin):ti,ab,kw OR (cefepime):ti,ab,kw OR (ceftazidime):ti,ab,kw | 2260 |
| #13 | (cefoperazone):ti,ab,kw OR (monobactam):ti,ab,kw OR (aztreonam):ti,ab,kw OR (ertapenem):ti,ab,kw OR (cefazolin):ti,ab,kw | 2204 |
| #14 | (clavulanic acid*):ti,ab,kw OR (sulbactam):ti,ab,kw OR (tazobactam):ti,ab,kw OR (beta-lactam*):ti,ab,kw | 3632 |
| #15 | #10 OR #11 OR #12 OR #13 OR #14 | 16225 |
| #16 | MeSH descriptor: [Drug Administration Schedule] explode all trees | 26086 |
| #17 | (extended):ti,ab,kw OR (prolonged):ti,ab,kw OR (continuous):ti,ab,kw OR (intermittent):ti,ab,kw | 237303 |
| #18 | #16 OR #17 | 257715 |
| #19 | MeSH descriptor: [Administration, Intravenous] explode all trees | 20378 |
| #20 | (infusion*):ti,ab,kw OR (administration*):ti,ab,kw | 429090 |
| #21 | #19 OR #20 | 429676 |
| #22 | #18 AND #21 | 93347 |
| #23 | #9 AND #15 AND #22 | 1885 |
| #24 | #9 AND #15 AND #22 Trials | 1861 |

3. Embase database search strategy（July 6, 2023）

| #1 | 'sepsis'/exp | 340532 |
| --- | --- | --- |
| #2 | 'septic shock'/exp | 71753 |
| #3 | 'systemic inflammatory response syndrome'/exp | 353222 |
| #4 | 'infection'/exp | 4496593 |
| #5 | sepsis:ab,ti OR 'septic shock':ab,ti OR bacteremia:ab,ti OR infection:ab,ti | 1912555 |
| #6 | #1 OR #2 OR #3 OR #4 OR #5 | 4914668 |
| #7 | 'beta lactam'/exp | 9793 |
| #8 | carbapenem:ab,ti OR penicillin:ab,ti OR piperacillin:ab,ti OR cephalosporin:ab,ti OR meropenem:ab,ti OR imipenem:ab,ti OR doripenem:ab,ti OR ticarcillin:ab,ti OR cefepime:ab,ti OR ceftazidime:ab,ti OR cefoperazone:ab,ti OR monobactam:ab,ti OR aztreonam:ab,ti OR ertapenem:ab,ti OR cefazolin:ab,ti OR 'clavulanic acid':ab,ti OR sulbactam:ab,ti OR tazobactam:ab,ti OR 'beta lactam':ab,ti | 154334 |
| #9 | #7 OR #8 | 159942 |
| #10 | 'drug administration'/exp | 1318570 |
| #11 | extended:ab,ti OR prolonged:ab,ti OR continuous:ab,ti OR intermittent:ab,ti | 1588280 |
| #12 | #10 OR #11 | 2823165 |
| #13 | 'intravenous drug administration'/exp | 400387 |
| #14 | infusion:ab,ti OR administration:ab,ti | 1534072 |
| #15 | #13 OR #14 | 1813233 |
| #16 | #12 AND #15 | 722772 |
| #17 | #6 AND #9 AND #16 AND ([randomized controlled trial]/lim OR 'controlled clinical trial'/de) AND [article]/lim AND [humans]/lim | 532 |
