# Supplementary figures and images for: Prolonged vs intermittent intravenous infusion of β-lactam antibiotics for patients with sepsis: a systematic review of randomized clinical trials with meta-analysis and trial sequential analysis
Source: Ann Intensive Care. 2023 Dec 5;13:121. doi: 10.1186/s13613-023-01222-w (PMC10697919; doi:10.1186/s13613-023-01222-w)

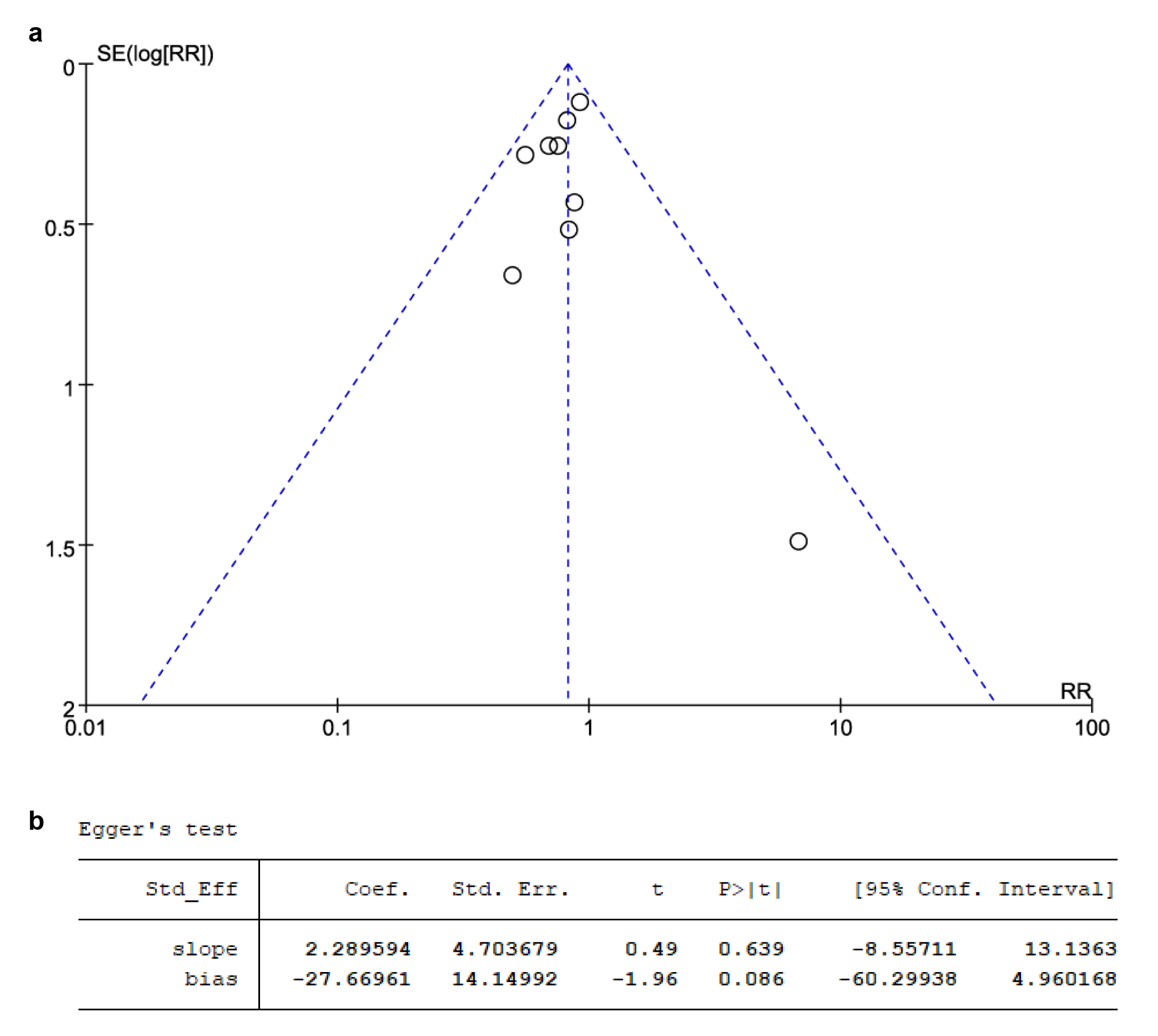

Supplement: Supplementary file 2 — Additional file 2. a. Funnel plots; b. Egger test. [file 13613_2023_1222_MOESM2_ESM.tif]

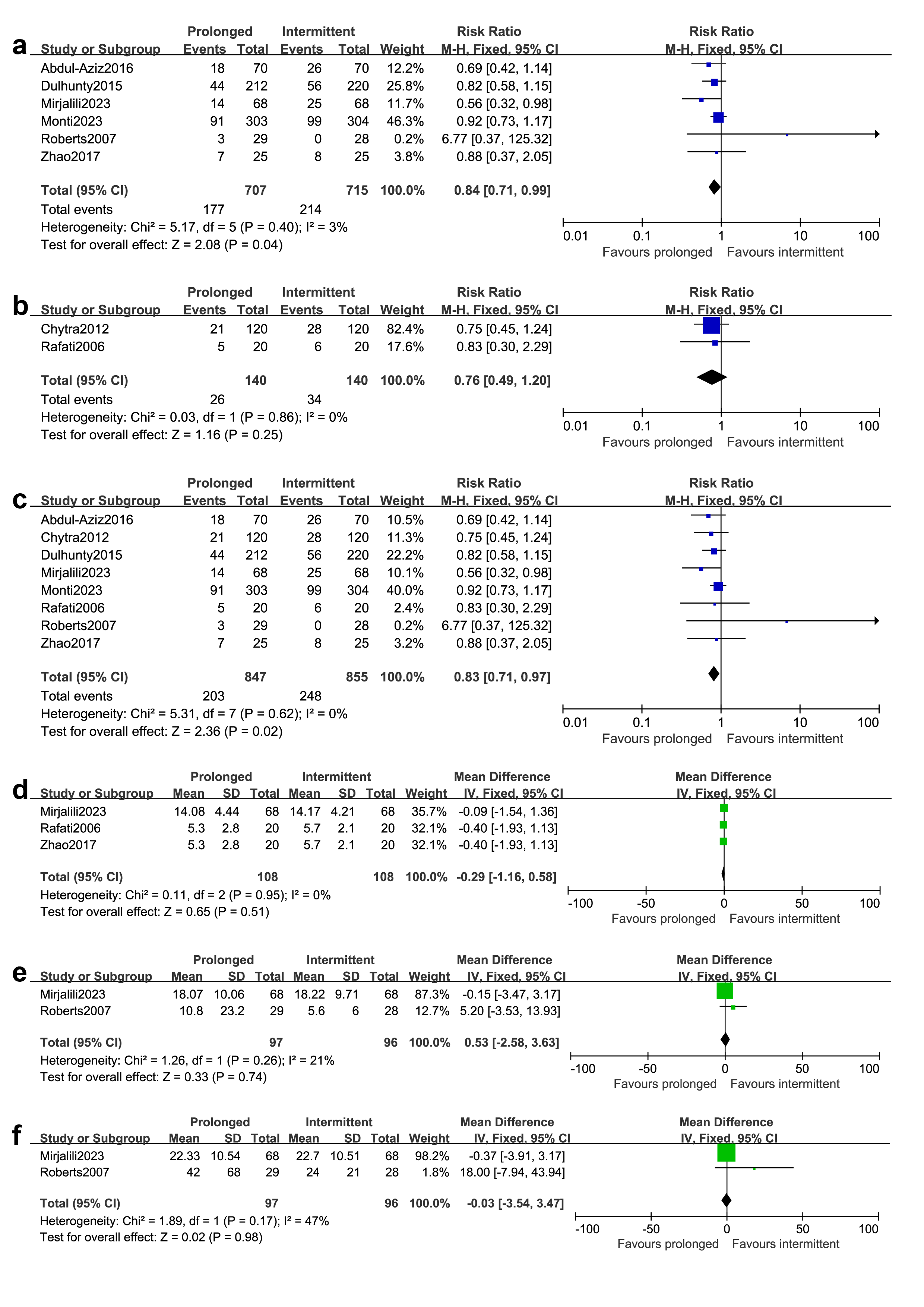

Supplement: Supplementary file 3 — Additional file 3. Forest plot of the subgroup analyses and sensitivity analyses: a. Subgroup analyses for the primary outcome based on doses of antibiotic (equal in two groups); b. subgroup analyses for the primary outcome based on doses of antibiotic (not equal in two groups); c. sensitivity analyses for the primary outcome by excluding one study without using of a loading dose in the prolonged infusion group; d. sensitivity analyses for duration of treatment; e. Sensitivity analyses for length of ICU stay; f. Sensitivity analyses for length of hospital stay. [file 13613_2023_1222_MOESM3_ESM.tif]
